# Neuropsychoanalysis: Bridging neuroscience and psychoanalysis to facilitate cultural attunement

**DOI:** 10.1371/journal.pmen.0000490

**Published:** 2025-11-11

**Authors:** Mohit Kumar, Sanjay Kumar, Khusboo, Vinit Kumar Singh, Amit Kumar Soni, Rounak Chatterjee

**Affiliations:** 1 Department of Psychiatry, All India Institute of Medical Sciences, Bhopal, India; 2 Department of Clinical Psychology, Institute of Mental Health Amritsar, Amritsar, India; 3 Department of Psychiatry, Chirayu Medical College & Hospital, Bhopal, India; 4 Department of Administration, All India Institute of Medical Sciences, Bhopal, India; 5 Department of Psychology, Government MLB Girls PG College, Kila Bhawan, Devi Ahilya University, Indore, India; PLOS: Public Library of Science, UNITED KINGDOM OF GREAT BRITAIN AND NORTHERN IRELAND

Neuropsychoanalysis, an interdisciplinary field bridging neuroscience and psychoanalysis, presents transformative opportunities for advancing mental health care globally. This Opinion considers its potential to address culturally specific psychological phenomena such as trance states and possession, which makes it especially promising for use in countries such as India. It also offers innovative frameworks for treating somatoform and trauma-related disorders. This Opinion bridges traditional mind–body–spirit paradigms with the principles of neuropsychoanalysis, advocating for their integration into therapeutic practices and academic research. While challenges such as limited interdisciplinary collaboration and lack of formal training persist globally, examples from India highlight both the opportunities and obstacles in applying these integrative frameworks within diverse cultural and clinical contexts. By addressing these barriers, neuropsychoanalysis can serve as a catalyst for culturally attuned and globally relevant innovations in mental health care and the scientific exploration of the human mind.

Neuropsychoanalysis has gained global prominence for its ability to address the complex interaction between neural mechanisms and unconscious processes. It bridges psychoanalytic understanding of affect, motivation, and defense with neuroscientific insights into brain networks involved in emotion regulation, memory, and self-reflection. For instance, the therapeutic exploration of unconscious conflicts can be viewed alongside neural mechanisms of the limbic and prefrontal systems involved in affect regulation. This integrative approach enhances psychotherapeutic practice by linking subjective experience with underlying neurobiological processes, thereby offering a more comprehensive understanding of the mind–brain relationship. [1]. Seminal works by scholars like Kandel (1999) have laid the foundation for understanding how the brain’s structures and functions interact with psychodynamic phenomena, influencing emotions, cognition, and behavior. In practice, Neuropsychoanalysis integrates neuroscientific insights into psychotherapeutic work helping clinicians understand how unconscious conflicts, affect regulation, and defense mechanisms are reflected in neural processes. For example, therapeutic attention to affective states can be informed by knowledge of limbic system functioning, while interventions aimed at enhancing reflective capacity may draw upon findings related to prefrontal cortical regulation. Thus, Neuropsychoanalysis bridges the subjective and biological dimensions of mental life in both conceptualization and clinical intervention. [2].

Despite notable advancements in neuroscience and a rich heritage of psychoanalytic thought, neuropsychoanalysis remains a largely underexplored domain in countries such as India. India’s unique socio-cultural framework, characterized by the centrality of community, spirituality, and family in shaping individual and collective psychology, offers fertile ground for applying neuropsychoanalytic perspectives. This approach can provide valuable insights into culturally embedded psychological constructs, such as the impact of familial bonds on mental health or the role of spiritual practices in emotional regulation and resilience.

Furthermore, integrating neuropsychoanalysis into India’s mental health research and clinical practices holds transformative potential. For instance, it could enhance the understanding of conditions like depression, anxiety, and trauma, where neural dysfunction and unconscious dynamics often interplay. By aligning these scientific advancements with cultural nuances and indigenous therapeutic practices, neuropsychoanalysis could significantly contribute to the holistic treatment of mental health conditions, addressing both the biological, psychological, and social dimensions of care, emphasizing the interdependence between neurobiological processes, intrapsychic dynamics, and the broader social environment that shapes individual functioning.

## The socio-cultural relevance of neuropsychoanalysis in India

India’s rich and diverse socio-cultural environment presents a unique and fertile landscape for neuropsychoanalytic research [3]. Traditional Indian philosophical systems, deeply rooted in ancient texts such as the Upanishads and the Bhagavad Gita, emphasize a holistic view of the individual, focusing on the interconnectedness of the mind, body, and spirit. This integrative approach aligns closely with the core objectives of neuropsychoanalysis, which seeks to bridge biological processes and psychodynamic experiences to form a cohesive understanding of human behavior and mental states [4].

In India, the emphasis on spirituality and its pervasive role in daily life offers a wealth of opportunities for neuropsychoanalysis to explore complex phenomena that transcend traditional psychoanalytic frameworks. Spiritual experiences, often described as profound states of consciousness or self-transcendence, remain difficult to explain solely through psychodynamic theories. Neuropsychoanalysis, by incorporating insights from neuroscience, provides a scientific basis for understanding such experiences. For instance, the study of brain activity during meditative states or religious rituals can reveal the neural underpinnings of altered states of consciousness, such as trance, possession, or the dissolution of ego boundaries [5]. These phenomena are particularly relevant in Indian cultural contexts, where spiritual practices like yoga, meditation, and devotional rituals are widespread and deeply embedded in the collective psyche.

Additionally, neuropsychoanalysis can contribute to understanding the mind-body connection, a concept integral to Indian traditional medicine systems like Ayurveda and Yoga. By examining how neural mechanisms interact with unconscious processes to influence physical health and emotional well-being, neuropsychoanalytic research can bridge ancient Indian wisdom with modern scientific paradigms. This could provide new insights into culturally specific mental health challenges, such as somatization disorders or the psychological dimensions of chronic illnesses, which are often framed within spiritual or holistic contexts in India.

Furthermore, the application of neuropsychoanalytic principles to Indian contexts can also illuminate the role of social structures, family dynamics, and community support systems in shaping mental health outcomes. The Indian cultural emphasis on collectivism, interdependence, and familial roles provides a rich backdrop for exploring how unconscious processes are influenced by societal expectations and relational patterns, thus deepening the understanding of both individual and collective mental health dynamics.

By integrating the scientific rigor of neuroscience with the depth of psychodynamic inquiry, neuropsychoanalysis has the potential to unlock a nuanced understanding of India’s socio-cultural and spiritual dimensions of mental health, thereby enriching global perspectives on the interplay between the brain, mind, and culture. Moreover, because neuropsychoanalysis does not contradict cultural or spiritual worldviews but rather provides a scientific lens through which these experiences can be understood, it may foster greater acceptance and adherence within communities that value holistic conceptions of the self and healing. [6].

## Therapeutic potential

Neuropsychoanalysis can advance mental health treatment in India by developing culturally sensitive therapeutic models. For example, by integrating psychoanalytic therapy with techniques like neurofeedback, clinicians could treat anxiety and post-traumatic stress disorder (PTSD) more effectively [7,8]. Affective neuroscience, which examines how the brain generates and regulates emotions, is especially relevant in the Indian context, where emotional experiences are deeply embedded within family, social, and cultural frameworks. Emotions in India are often shaped by close-knit family structures, communal expectations, and spiritual beliefs, which influence how feelings are expressed, experienced, and managed. By studying the neural mechanisms underlying emotional regulation in these culturally grounded contexts, affective neuroscience can help explain why certain individuals may be more vulnerable to mood and anxiety disorders. Moreover, this understanding can guide the development of culturally sensitive interventions that resonate with local beliefs and practices, enhancing their effectiveness and acceptance. [9,10].

## Research opportunities

The research potential of neuropsychoanalysis in India is vast, particularly in investigating culturally and spiritually significant phenomena. One promising avenue is the neurobiological study of trance, possession states, and ritual practices, which are prevalent in many Indian traditions. These experiences, often considered spiritual or mystical, may involve distinct patterns of neural connectivity and activation that can be explored using advanced neuroimaging techniques such as functional MRI (fMRI), diffusion tensor imaging (DTI), and magnetoencephalography (MEG) [11,12]. Such studies could help bridge the gap between subjective cultural experiences and objective neural mechanisms, providing a scientifically grounded understanding of phenomena traditionally explored only in anthropological or psychological contexts.

In addition, the integration of machine learning with psychoanalytic frameworks offers a novel methodological frontier. By analyzing complex neural data collected during psychotherapy, machine learning models could detect subtle patterns corresponding to unconscious processes, emotional shifts, or therapeutic breakthroughs. This approach allows for the quantification of phenomena that have traditionally been considered inaccessible to objective measurement, offering new ways to assess therapeutic progress and refine psychotherapeutic interventions.

Taken together, these avenues highlight how neuropsychoanalysis in India can uniquely combine cultural, spiritual, and neuroscientific perspectives, opening opportunities for research that is both scientifically rigorous and culturally resonant. [13,14].

## Tackling challenges in integrating neuropsychoanalysis in India

Despite its potential, several obstacles hinder the growth of neuropsychoanalysis in India. A major challenge is the lack of interdisciplinary collaboration between neuroscientists and psychoanalysts. Furthermore, neuropsychoanalysis is not widely taught in postgraduate psychology programs, limiting awareness among future clinicians and researchers. Funding and infrastructural limitations further impede the establishment of neuropsychoanalytic research centers [15,16].

Integrating neuropsychoanalysis into India’s mental health care system requires advocacy at multiple levels. Introducing neuropsychoanalytic modules in psychology and medical curricula can equip students with the skills needed for interdisciplinary research [2]. Workshops and conferences focused on neuropsychoanalysis can stimulate professional interest and encourage collaboration between psychologists, psychiatrists, and neuroscientists [17,18].

## Conclusion

Neuropsychoanalysis offers profound potential for transforming mental health care in India by merging neuroscientific and psychodynamic approaches. It holds the promise of providing holistic, culturally relevant therapeutic interventions while advancing research on the neural mechanisms underlying psychological phenomena. However, realizing this potential requires overcoming significant barriers, including limited collaboration and insufficient training opportunities. By fostering interdisciplinary dialogue, securing funding, and integrating neuropsychoanalysis into academic and clinical practice, India can lead innovative developments in global neuropsychoanalytic research.
